# Re-resection in Incidental Gallbladder Cancer: Survival and the Incidence of Residual Disease

**DOI:** 10.1245/s10434-019-08074-4

**Published:** 2019-11-18

**Authors:** Elise A. J. de Savornin Lohman, Lydia G. van der Geest, Tessa J. J. de Bitter, Iris D. Nagtegaal, Cornelis J. H. M. van Laarhoven, Peter van den Boezem, Chella S. van der Post, Philip R. de Reuver

**Affiliations:** 1 Department of Surgery, Route 618, Radboudumc, Nijmegen, The Netherlands; 2 Netherlands Comprehensive Cancer Organization, Utrecht, The Netherlands; 3 Department of Pathology, Radboudumc, Nijmegen, The Netherlands

## Abstract

**Background:**

Re-resection for incidental gallbladder cancer (iGBC) is associated with improved survival but little is known about residual disease (RD) and prognostic factors. In this study, survival after re-resection, RD, and prognostic factors are analyzed.

**Methods:**

Patients with iGBC were identified from the Netherlands Cancer Registry, and pathology reports of re-resected patients were reviewed. Survival and prognostic factors were analyzed.

**Results:**

Overall, 463 patients were included; 24% (*n* = 110) underwent re-resection after a median interval of 66 days. RD was present in 35% of patients and was most frequently found in the lymph nodes (23%). R0 resection was achieved in 93 patients (92%). Median overall survival (OS) of patients without re-resection was 13.7 (95% confidence interval [CI] 11.6–15.6), compared with 52.6 months (95% CI 36.3–68.8) in re-resected patients (*p* < 0.001). After re-resection, median OS was superior in patients without RD versus patients with RD (not reached vs. 23.1 months; *p* < 0.001). In patients who underwent re-resection, RD in the liver (hazard ratio [HR] 5.54; *p* < 0.001) and lymph nodes (HR 2.35; *p* = 0.005) were the only significant prognostic factors in multivariable analysis. Predictive factors for the presence of RD were pT3 stage (HR 25.3; *p* = 0.003) and pN1 stage (HR 23.0; *p* = 0.022).

**Conclusion:**

Re-resection for iGBC is associated with improved survival but remains infrequently used and is often performed after the optimal timing interval. RD is the only significant prognostic factor for survival after re-resection and can be predicted by pT and pN stages.

**Electronic supplementary material:**

The online version of this article (10.1245/s10434-019-08074-4) contains supplementary material, which is available to authorized users.

Gallbladder cancer (GBC) is the most prevalent biliary tract malignancy and the sixth most common gastrointestinal malignancy worldwide.^1^ Due to an asymptomatic course in the early stages, patients are frequently diagnosed in an advanced stage and prognosis is extremely poor.^2^^–^5 However, long-term survival does occur in patients with early-stage tumors.^6^ These patients are most frequently diagnosed incidentally (iGBC), after cholecystectomy for presumed benign gallbladder disease.^7^^–^9 Due to the growing number of laparoscopic cholecystectomies performed, iGBC is an increasingly relevant clinical issue.^10^^,^^11^ Noticeably, especially in the Western world, many GBCs are detected as an incidental finding.^2^^,^^12^

In order to prevent early locoregional recurrence, re-exploration and definitive resection is currently recommended for patients with tumors invading the muscle layer and no evidence of disseminated disease.^10^ Re-resection involves a partial hepatectomy of segments 4b/5, either as a full segmentectomy or wedge excision, and resection of the hepatoduodenal lymph nodes.^13^

Re-resection is associated with improved survival in retrospective studies. However, it is still controversial whether resecting residual disease (RD) actually improves survival or whether it merely enables more complete staging and consequently provides more accurate estimation of survival.

Prognosis after re-resection appears to be primarily determined by the presence of RD and lymph node metastases.^6^,^14^^–^16 Interestingly, although the likelihood of detecting RD increases concurrently with T stage, a study including 135 patients found that survival did not differ between T2 and T3 tumors in patients in whom no RD was detected.^15^ This finding suggests that rather than T stage, the presence of RD after re-resection appears to be the primary predictor for survival.

Evidently, identifying patients at risk for RD after re-resection could greatly improve candidate selection for additional surgery. Patients who are likely to have RD could potentially benefit from more aggressive surgery. On the other hand, in patients at low risk of RD, a more conservative approach could be justified.

The aim of this study was to assess survival of iGBC patients with and without re-resection. Second, we assessed the prognostic value of histopathological characteristics on survival after re-resection.

## Methods

This was a retrospective, nationwide cohort study that was approved by the NCR Ethical Review Board. A waiver for ethical approval was provided by the Medical Ethics Review Committee of the Arnhem–Nijmegen region (CMO A-N, nr. 2017-3912), and the Strengthening the Reporting of Observational Studies in Epidemiology (STROBE) statement for reporting of observational cohort studies was followed.^17^

### Patient Selection and Variable Definitions

All patients diagnosed with iGBC from 2000 to 2016 were identified from the Netherlands Cancer Registry (NCR). The NCR contains data on all newly diagnosed malignancies, including year of diagnosis, patient age and gender, and tumor characteristics (cTNM and pTNM stage).^18^ Notification sources were the nationwide network and registry of histopathology and cytopathology in The Netherlands (PALGA^19^) and data from the National Registry of Hospital Discharge Diagnoses. Follow-up data on vital status (complete until February 2018) were provided by linkage to the automated Municipal Personal Records Database. iGBC was defined as GBC diagnosed based on postoperative histopathological examination. All patients with pre- or perioperative suspicion of GBC (defined as suspicion of GBC on preoperative imaging or findings suspect for malignancy during surgery) were excluded since the NCR categorizes these patients as suspected GBC. Patients with T1a disease or metastatic disease (detected by imaging during postoperative re-staging or during re-exploration within 6 months of diagnosis) were excluded from analysis since these patients have no indication for additional radical surgery.

Re-resection was defined as any additional, GBC-directed surgery within 6 months after the primary surgery. A retrospective review of the complete pathology reports of re-resected patients was performed using data supplied by PALGA. For patients who received a re-resection, the pTNM stage as reported after primary surgery was used to reconstruct the initial TNM stage. Because the location of the tumor was frequently not reported, no differentiation between serosal and liver side tumors could be made, and all tumors were classified according to the 7th edition of the American Joint Committee on Cancer (AJCC) staging manual.^18^ Adjuvant chemo(radio)therapy is not considered standard of care in The Netherlands and was not administered to any of the patients throughout the study period.

For re-resected patients for whom complete histopathological reports were available, the following variables were extracted from the primary surgery report: type of surgery (laparoscopic cholecystectomy, open cholecystectomy, other, unspecified), pTNM stage, tumor size, tumor differentiation, presence of perineural/perivascular/lymphatic growth, and radicality (R0 defined as no microscopically present tumor < 1 mm from the resection margin). The following variables were assessed in the re-resection report: cystic duct stump resection (yes/no), lymphadenectomy (yes [number of lymph nodes resected]/no), liver resection (no/gallbladder bed/one segment/two segments/≥ 3 segments), presence and location of RD (defined as findings of microscopic liver/lymph node/cystic duct involvement in the pathological examination after radical surgery) and radicality of the re-resection.

### Statistical Analysis

Patient and tumor characteristics were described using counts and percentages for categorical variables, and means and ranges for continuous variables. Patients who underwent re-resection were categorized as having T1b, T2, or T3/Tx disease, based on the T stage after primary resection (no patients with T4 disease received a re-resection). All analyses for patients with a re-resection were conducted using the T stage as assessed after the primary resection. The Chi square or Fisher’s exact tests were used, where appropriate, to assess differences in the extent of re-resection performed and the presence and location of RD, and Kaplan–Meier curves were used to calculate the median survival times. Survival was defined as the time in days from the date of diagnosis (primary surgery) until the date of death from any cause or the date of end of follow-up.^20^ Log-rank testing and Cox regression analysis were used to compare survival between groups of patients. To deal with immortal time bias of patients who underwent re-resection, patients with a follow-up duration of < 90 days after resection were excluded for all comparative survival analyses. Additionally, to reduce treatment selection bias in the calculation of median survival times, the Kaplan–Meier method was repeated in patients under 65 years of age.

Cox regression analysis was used to calculate hazard ratios (HRs) for potential prognostic factors in patients who underwent re-resection, and logistic regression was used to identify factors predictive for RD. Covariates were selected based on the literature and were entered in the multivariable model when statistically relevant (*p* < 0.1) on univariable analysis. A stepwise forward selection approach was used. Missing data were determined to be ‘missing at random’ (unrelated to the outcome, potentially related to other parameters) and complete case analysis was used to assess covariates.^20^,^21^

*p* values < 0.05 were considered statistically significant, and all tests of significance were two-tailed. Statistical analyses were conducted using the SPSS 25.0 statistical package (IBM Corporation, Armonk, NY, USA).

## Results

### Patient and Tumor Characteristics

**Fig. 1:**
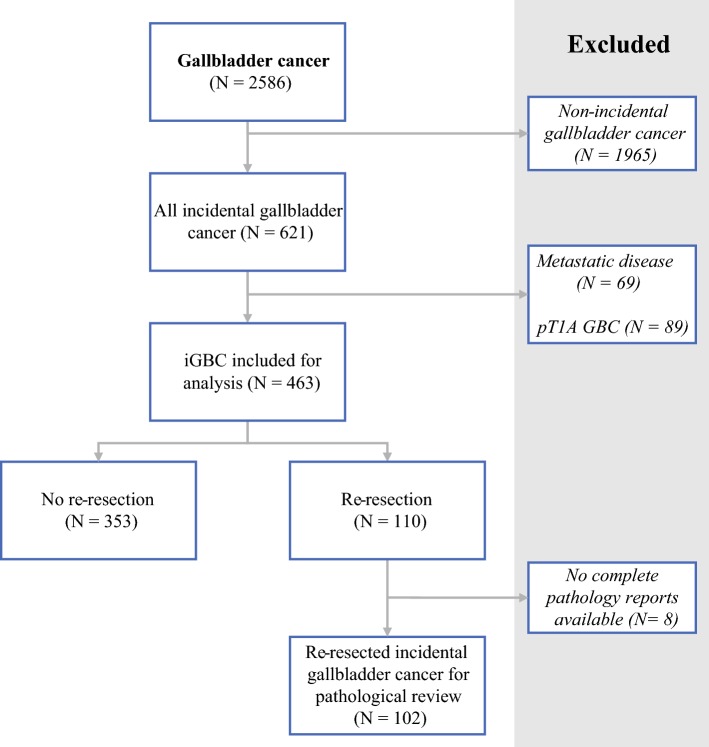
Selection of the included patients. *GBC* gallbladder cancer, *iGBC* incidental gallbladder cancer

A total of 463 patients with iGBC were included (Fig. 1), of whom 110 patients (23%) underwent re-resection. Patient and tumor characteristics are displayed in Table 1. Patients with a re-resection were significantly younger; the mean age difference was 10 years and 43% of patients ≤ 65 years of age received a re-resection, as opposed to 15% of patients aged 66 years or older (*p* < 0.001). Furthermore, re-resected patients were more likely to have T2 disease (66% vs. 49%; *p* = 0.020) and node-positive disease (12% vs. 6%; *p* = 0.001).

**Table 1:** Baseline patient and tumor characteristics

	Re-resection (*N* = 110)	No re-resection (*N* = 353)	*p* value
Age, years [mean (range)]	62.9 (36–81)	72.2 (25–97)	**<0.001**
Sex, male	33 (30.0)	93 (26.3)	0.452
Tumor differentiation grade			
Well	16 (14.5)	38 (10.8)	0.244
Moderately	47 (40.9)	124 (35.1)	
Poor	22 (20.0)	101 (28.6)	
Unknown	27 (24.5)	90 (25.5)	
pT stage			
T1b	10 (9.1)	47 (13.3)	0.079
T2	74 (67.3)	185 (52.4)	
T3/T4	24 (21.8)	90 (25.5)	
T*x*	2 (1.8)	31 (8.8)	
pN stage			
N0	22 (20.0)	132 (37.4)	**0.001**
N1–2	13 (11.8)	22 (6.2)	
N*x*	75 (68.2)	199 (56.4)	
Resection margin			
R0	73 (66.4)	160 (45.3)	**<0.001**
R1/R2	32 (29.1)	95 (26.9)	
Unknown	5 (4.5)	98 (27.8)	

Data are expressed as *n* (%) unless otherwise specified

Bolded values indicate statistical significance (*P* < 0.005)

### Re-resection Procedures and Histopathology Assessment

**Fig. 2:**
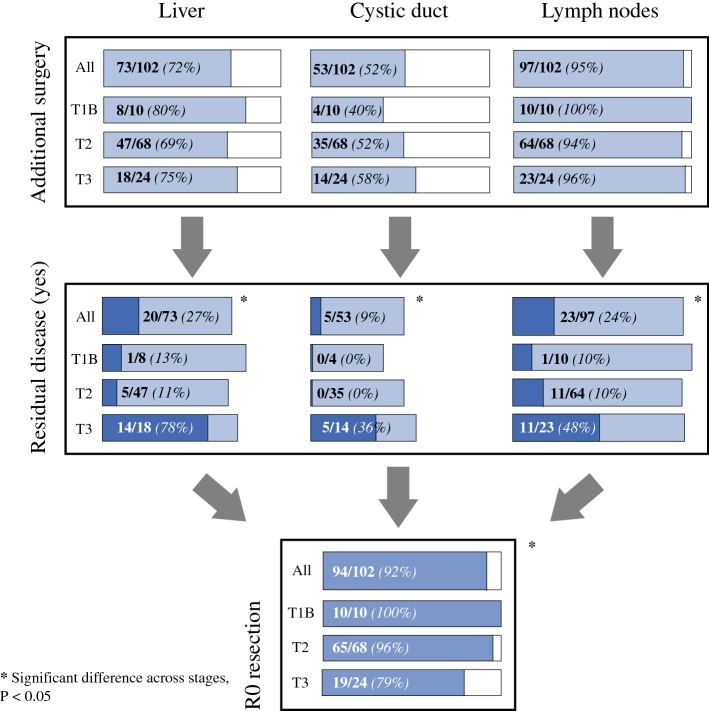
Extent of resection and incidence of residual disease according to T stage

Complete histopathology reports were available in 102 patients who underwent re-resection. Primary surgery of these 102 patients was laparoscopic cholecystectomy in 26 (25%) patients, open cholecystectomy in 4 (4%) patients, subtotal cholecystectomy in 6 (6%) patients, and unspecified in 66 (65%) patients. The median interval between primary surgery and re-resection was 66 days [interquartile range (IQR) 47–83]. An overview of the re-resection procedures conducted and the incidence of RD is provided in Fig. 2. Ninety-seven patients underwent dissection of the hepatoduodenal ligament, with a median lymph node harvest of 3 (range 2–20). Seventy-three patients underwent resection of the liver parenchyma; gallbladder bed resection in 55 (75%) patients, gallbladder bed resection plus resection of segments 4 and 5 in 17 (23%) patients, and right hemihepatectomy in 1 (1%) patient. Fifty-three (52%) patients underwent resection of the cystic duct stump, of whom 8 (8%) also underwent extrahepatic bile duct resection.

No significant differences in the extent of resection was found between T stages (Fig. 2). RD was significantly more present in re-resection specimens of patients with T3 disease. R0 re-resection was achieved in 92% of patients across the re-resected cohort, but in only 72% of patients with T3 disease (*p* < 0.001).

### Survival in Incidental Gallbladder Cancer

**Fig. 3:**
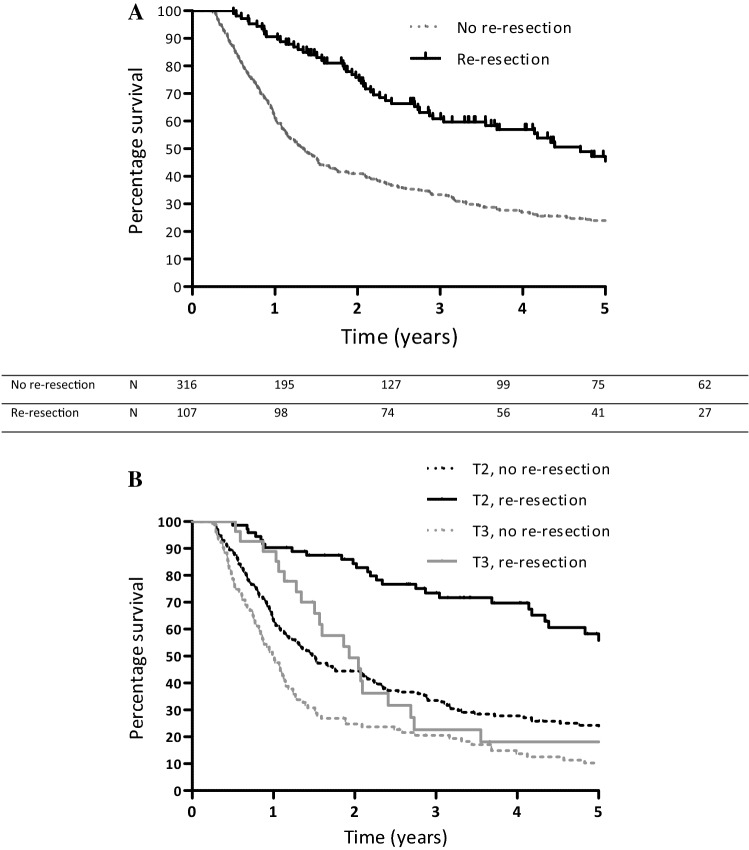
**a** Overall survival of patients with iGBC (*N* = 423 after exclusion of follow-up of < 90 days), by re-resection (yes, no). Log-rank *p* < 0.001. **b** Overall survival of patients with T2 (*N* = 243) and T3/T*x* (*N* = 130) iGBC after exclusion of follow-up of < 90 days, by re-resection. Log-rank *p* < 0.001. *iGBC* incidental gallbladder cancer

The median follow-up in the entire cohort (*n* = 463) was 17.8 months (IQR 8.1–36.7), and median overall survival (OS) was 18.3 months [95% confidence interval (CI) 14.1–22.4]. Median OS of iGBC patients without re-resection was 13.7 (95% CI 11.6–15.6), compared with 52.6 months (95% CI 36.3–68.8) in patients who underwent re-resection (*p* < 0.001). When patients with a follow-up duration of < 90 days from the primary surgery were excluded from the analysis, survival was 16.1 months (95% CI 13.7–18.5) in patients without re-resection and 56.3 months (95% CI 49.0–63.5) in re-resected patients (*p* < 0.001) (Fig. 3a). When selecting patients under the age of 65 years and with a follow-up duration of ≥ 90 days, re-resection was still associated with superior survival (18 vs. 77 months; *p* < 0.001).

In multivariable analysis including patients with ≥ 90 days of follow-up, and controlling for age, T stage, nodal status, resection margin and tumor grade, re-resection remained a significant predictor for superior survival (HR 0.464, 95% CI 0.338–0.639; *p* < 0.001) [electronic supplementary Table 1].

In a subgroup analysis of all patients who had tumor-free resection margins at the primary resection (*N* = 226) median OS was 25.9 months in patients without re-resection (95% CI 14.3–37.5) versus 83.8 months (95% CI 41.6–125.9) in patients who received a re-resection (*p* < 0.001). After excluding patients with < 90 days of follow-up, this difference persisted: 28.2 months (R0) versus 90.0 months (R1) (*p* < 0.001). Median OS (after exclusion of patients with a follow-up duration of < 90 days) in re-resected T2 disease was 60.0 months, versus 18.1 months (*p* < 0.001) in non-re-resected T2 disease. Median OS in re-resected T3 disease was 23.1 months versus 12.1 months (*p* < 0.015) in non-re-resected T3 disease (Fig. 3b). Re-resection in T1b iGBC was not significantly associated with longer survival (median OS re-resected T1b = 56.0 months vs. no resection T1b = 60.0 months; *p* = 0.705).

### Prognostic Factors and Survival After Re-resection

**Fig. 4:**
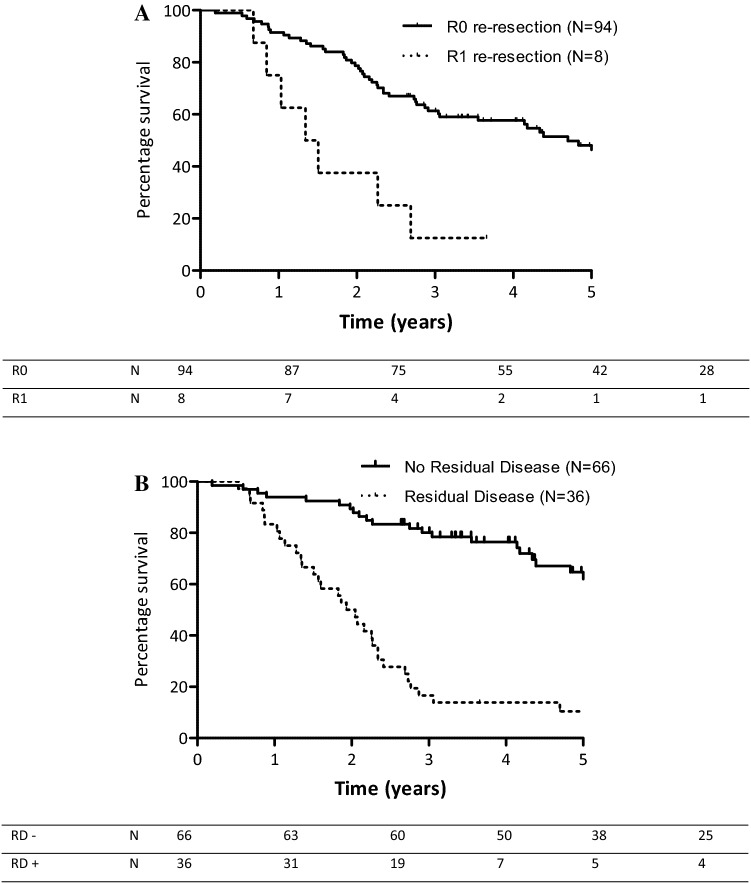
Overall survival of patients with iGBC after re-resection (*N* = 102), by **a** margin status and **b** residual disease. Log-rank *p* < 0.001. *iGBC* incidental gallbladder cancer, *RD* residual disease

In patients who received a re-resection and for whom complete pathology reports were available (*n* = 102), median OS was 56.3 months (95% CI 32.3–80.2) in patients with tumor-free resection margins versus 18.0 months (95% CI 13.1–23.0) in patients with tumor-positive resection margins in re-resection specimens (*p* < 0.001) (Fig. 4a). No significant survival difference was seen between patients who did and did not receive any form of liver resection (i.e. gallbladder bed, segmentectomy, or hemihepatectomy), neither across the entire cohort (50.0 vs. 52.6 months; *p* = 0.601) nor stratified according to T stage (data not shown). Median OS in patients without RD (*N* = 66) in the re-resection specimen was not reached, versus 23.1 months (95% CI 18.8–27.5) in patients in whom RD was present (*N* = 36; *p* < 0.001) (Fig. 4b). No survival differences were found between different locations of RD; patients with RD in the liver only (*n* = 13) had a median OS of 22.9 months, versus 24.5 months in patients with RD in the lymph nodes alone (*n* = 16) and 22.3 months in patients with RD in both liver and lymph nodes (*n* = 7) (*p* = 0.257). In patients with RD, no significant difference in median OS was found between patients with tumor-free resection margins (R0, *n* = 8; median OS 24.5 months) in the re-resection specimen and patients in which re-resection margins were not clear (R1, *n* = 28; median OS 16.1 months) (*p* = 0.447).

On univariable screening, significant prognostic factors associated with worse outcome after re-resection were pT3 stage, irradical (R1/R2) resection margins after re-resection, perineural and lymphovascular invasion, and the presence of RD in the lymph nodes, cystic duct, and liver (Table 2). In the multivariable Cox proportional hazards model, only the presence of RD in the lymph nodes (HR 2.35; *p* = 0.005) or liver (HR 5.54; *p* < 0.001) remained significant prognostic factors (Table 2).

**Table 2:** Prognostic factors for survival after re-resection in patients with incidental gallbladder cancer (*N* = 102)

Characteristic	Univariable cox regression			Multivariable cox regression		
	HR	95% CI	*p* value	HR	95% CI	*p* value
Age, years	1.02	0.99–1.05	0.156			
Pathological N stage						
N0	1					
N1/N2	0.72	0.38–1.35	0.303			
N*x*	1.38	0.80–2.37	0.247			
Pathological T stage						
T1	1			^c^		
T2	1.42	0.50–4.01	0.512	^c^		
T3/T*x*	4.09	1.39–12.04	0.011	^c^		
Radicality re-resection						
R0	1			^c^		
R1/R2	3.93	1.74–8.88	0.001	^c^		
Tumor differentiation grade						
Well	1					
Moderate	0.81	0.37–1.78	0.606			
Poor	1.20	0.52–2.80	0.668			
Unknown	0.82	0.34–1.95	0.648			
Residual disease, lymph node (yes)	3.18	1.84–5.52	< 0.001	**2.35**	**1.30–4.23**	**0.005**
Residual disease, liver (yes)	7.08	3.57–14.05	< 0.001	**5.54**	**2.70–11.37**	**<0.001**
Residual disease, cystic duct (yes)	5.82	2.17–15.57	< 0.001	^c^		
Lymphovascular invasion (yes)^a^	2.31	1.36–3.91	0.002	^c^		
Perineural invasion (yes)^b^	1.86	1.06–3.27	0.031	^c^		

All variables with *p* < 0.10 on univariable analysis were entered into the multivariable model

Bolded values indicate statistical significance (*P* < 0.005)

*HR* hazard ratio, *CI* confidence interval

^a^Missing values in 10 cases

^b^Missing values in 13 cases

^c^Not significant during forward selection

### Predictive Factors for Residual Disease

On univariable screening, N1 disease, T3 disease, R1/R2 resection margins at primary cholecystectomy, lymphovascular invasion, and perineural invasion were predictive for RD in re-resected patients (Table 3). When entered into a multivariable model, only N1 (HR 23.0; *p* = 0.022) and pT3 disease (HR 25.3; *p* = 0.003) remained predictive of the presence of RD.

**Table 3:** Predictive factors for the presence of residual disease after re-resection in patients with incidental gallbladder cancer (*n* = 102)

Characteristic	Univariable logistic regression			Multivariable logistic regression		
	HR	95% CI	*p* value	HR	95% CI	*p* value
Tumor differentiation grade						
Well	1			^c^		
Moderate	2.49	0.62–9.96	0.197	^c^		
Poor	5.03	1.17–21.59	0.030	^c^		
Unknown	1.56	0.26–9.47	0.632	^c^		
Pathological N stage						
0	1			1		
1/2	25.00	2.36–264.80	0.008	23.00	1.57–337.44	0.022*
Unknown	1.00	0.284–3.53	1.000	1.053	0.14–4.18	0.763
Pathological T stage						
T1	1			1		
T2	0.93	0.20–5.44	0.966	1.22	0.18–8.52	0.838
T3/T*x*	11.60	3.04–131.73	0.002	25.33	2.98–215.69	0.003*
Time since index surgery (days)	1.00	0.99–1.01	0.788			
Radicality primary resection						
R0	1			^c^		
R1/R2	4.80	1.97–11.70	0.001	^c^		
Unknown	1.14	0.11–11.87	0.911	^c^		
Socioeconomic status (deciles)	1.03	0.88–1.19	0.743			
Hospital of diagnosis						
Community	1					
Academic	0.21	0.03–1.73	0.146			
Lymphovascular invasion (yes)^a^	4.25	1.77–10.18	0.001	^c^		
Perineural invasion (yes)^b^	3.18	1.25–8.09	0.015	^c^		

All variables with *p* < 0.10 on univariable analysis were entered into the multivariable model

^a^Missing values in 10 cases

^b^Missing values in 13 cases

^c^Not significant during forward selection

*p value < 0.005

## Discussion

The present study demonstrates that re-resection was associated with increased survival in patients with T2 and T3 iGBC, and that re-resection remained an independent favorable prognostic factor in multivariable analysis. In patients who underwent re-resection, RD was more often found in patients with a higher pT stage, and the presence of RD was the primary determinant of worse survival.

Although international guidelines recommend radical cholecystectomy for all iGBC patients, except those with T1a disease, the management of T1b iGBC remains controversial. Results from the literature are conflicting; some studies do not report a survival benefit,^22^^–^24 whereas other series show an increase in 5-year survival of up to 30% after radical cholecystectomy.^25^,^26^ Interestingly, although a general survival benefit was shown across the entire re-resected cohort, patients with T1b disease did not show superior survival after re-resection. Potentially, re-resection in T1b disease is not beneficial due to the low prevalence of RD; only 1 of 10 T1b patients had RD. Another explanation might be that the extent of surgery in these patients was too small to provide a survival benefit; in all T1b patients, only a 1–2 cm, non-anatomic wedge resection of the gallbladder bed was performed and median lymph node harvest was only 2. This may have resulted in understaging and undertreatment and masked the potential benefit of radical cholecystectomy. Finally, our cohort was potentially too small to detect a significant difference in survival.

On the other hand, re-resection for T3 patients is currently not considered standard practice in the Dutch national guideline due to a lack of perceived benefit.^27^ In our cohort, median OS was 1 year in patients without re-resection and 1.9 years in re-resected patients with T3 disease (landmark at 90 days). Four of 24 (17%) patients with T3 disease had tumor-free margins at primary surgery. After re-resection, tumor-free margins were achieved in 19/24 patients (79%). Survival in GBC is primarily determined by the ability to achieve tumor-free margins;^10^ therefore, the increased rate of R0 resections after re-resection is the most likely cause for the higher survival in re-resected T3 disease.

International guidelines recommend re-resection for all patients with ≥ T1b iGBC fit to undergo surgery within 4–8 weeks from the initial cholecystectomy.^13^,^28^ Worrisomely, in our cohort, only 23% of patients received a re-resection and the median time interval between index surgery and re-resection was over 9 weeks. In a recent publication from Sweden, 121/201 (60%) non-metastatic iGBC patients received a re-resection.^24^ In 27 (13%) of all patients, a re-resection was not performed due to comorbidities. Another study included 218 iGBC patients and re-resection was attempted in 188 (86%) patients.^16^ Only 17 (8%) patients did not undergo a re-resection due to low performance status. Unfortunately, due to the nature of our study, we were not able to assess the rationale for not performing a re-resection in our cohort; however, it is evident from other studies that comorbidities do not frequently preclude re-resection. Other factors such as physician unawareness of or ambiguity regarding the efficacy of re-resection may account for the low number of re-resected patients in our cohort.

Moreover, our results show considerable practice variation regarding the extent of re-resection performed. International guidelines recommend gallbladder bed resection for all patients, as well as lymphadenectomy with a minimum count of six nodes.^13^ In our cohort, only 72% of patients received any form of liver resection and the median lymph node harvest was three. Evidently, guideline adherence is suboptimal and more extensive surgery than is currently performed is necessary to improve outcomes.

Additionally, our results raise concerns on the accuracy of staging of iGBC, especially in T2 disease. In the case of two-stage procedures, it is impossible to differentiate between metastatic disease and underestimation of T stage at initial assessment.

RD was found in 35% of patients who underwent re-resection and was most frequently located in the lymph nodes (23%) and liver (20%). RD in the extrahepatic bile duct was found in four of eight patients (50%). These findings conflict recent literature, in which a higher rate (up to 60%) of RD was found.^6^,^16^ However, our finding that RD is mostly found in T3 disease and (regardless of site) is the primary determinant of survival after re-resection is in line with previously published literature.

The finding that RD is the primary determinant of survival raises questions about the value of re-resection in iGBC. The goal of re-resection is to clear the patient of residual local or regional disease and consequently improve survival. However, survival in patients in which RD is found is poor, even when resection margins are clear. Moreover, no significant survival difference was found between patients with RD who received R0 versus R1 re-resection. This contradicts the notion that the increase in survival seen after re-resection stems from complete tumor clearance. Potentially, re-resection is beneficial solely for patients in which only microscopic RD, undetected by the pathologist, is present. When macroscopic RD is found, the tumor may have already progressed beyond potential curation. The fact that survival between patients with different locations of RD did not differ suggests that the presence of RD acts as the clinical and prognostic equivalent of metastatic disease.

The fact that patients with RD appear unlikely to benefit from surgical treatment alone gives rise to novel clinical challenges. Although data are lacking, patients with iGBC may benefit from (neo)adjuvant chemotherapy, especially when RD is present. Predicting which patients are likely to have RD could be a useful tool to identify potential candidates for neoadjuvant treatment. Perineural and lymphovascular invasion, R1/R2 margins at initial cholecystectomy, tumor grade, and pT and pN stage were univariably associated with the presence of RD in our cohort. After multivariable analysis, only pT and pN stage appeared predictive for RD, although CIs were wide. The other factors may have remained significant if more patients were included. Two studies with larger cohorts produced similar results;^29^,^30^ however, CIs were either not reported or were very wide, and pN stage was not included in their models. Future, larger cohorts are needed to further identify histopathological characteristics associated with RD.

This study has several limitations. Primarily, our results are sensitive to selection bias due to the retrospective study design. For example, improved survival after re-resection in T3 disease may very well be a result of treatment selection bias and immortal time bias rather than a potential therapeutic effect of re-resection. We attempted to address these biases by landmarking, multivariable analysis, and subgroup analysis in younger patients; however, some bias may still be present. Second, pathology reports were reviewed but no revision of the actual resection specimens was performed. Review by an expert hepatobiliary pathologist may have altered our results. Finally, survival according to T stage in non-re-resected patients may have been underestimated due to understaging.

A strength of this study is that our results are based on actual nationwide outcomes, and generalizability is therefore likely high. Moreover, our study is the only study that used landmark and stratification techniques when investigating the value of re-resection in iGBC, thus reducing the effects of the aforementioned biases.

## Conclusion

There is substantial surgical undertreatment for iGBC in The Netherlands; re-resection is associated with improved survival in T2 and T3 iGBC. The presence of RD is the main prognostic factor for survival after re-resection and can be predicted by pT and pN stage. Additional histopathological research is necessary to identify candidates most likely to benefit from additional surgery and possible neoadjuvant chemotherapy.

## Electronic supplementary material

Below is the link to the electronic supplementary material.

Supplementary material 1 (DOCX 13 kb)
