# Supplementary material for: Re-resection in Incidental Gallbladder Cancer: Survival and the Incidence of Residual Disease
Source: Ann Surg Oncol. 2019 Nov 18;27(4):1132–42. doi: 10.1245/s10434-019-08074-4 (PMC7060151; doi:10.1245/s10434-019-08074-4)
Supplement: Supplementary file 1 — Supplementary material 1 (DOCX 13 kb) [file 10434_2019_8074_MOESM1_ESM.docx]

**Supplementary Table 1.**

|  | **Univariable cox regression** | | | **Multivariable cox regression** | | |
| --- | --- | --- | --- | --- | --- | --- |
| **Characteristic** | **HR** | **95% CI** | **P value** | **HR** | **95% CI** | **P value** |
| Age (year) | 1.03 | 1.02 – 1.04 | <0.001 | **1.02** | **1.01 – 1.03** | **<0.001** |
| Re-resection (yes) | 0.43 | 0.32 – 0.58 | <0.001 | **0.47** | **0.34 – 0.65** | **<0.001** |
| Pathological T stage |  |  |  |  |  |  |
| T1 | 1 |  |  | 1 |  |  |
| T2 | 1.47 | 0.99 – 2.18 | 0.057 | 1.45 | 0.97 – 2.16 | 0.069 |
| T3/T4 | 2.84 | 1.89 – 4.28 | <0.001 | **2.14** | **1.39 – 3.28** | **0.001** |
| Pathological N-stage |  |  |  |  |  |  |
| N0 | 1 |  |  | 1 |  |  |
| N1 | 3.85 | 2.15 – 6.91 | <0.001 | 2.53 | **1.39 – 4.62** | **0.002** |
| Nx | 3.31 | 1.98 – 5.57 | <0.001 | 2.23 | **1.31 – 3.81** | **0.003** |
| Radicality primary resection |  |  |  |  |  |  |
| R0 | 1 |  |  | 1 |  |  |
| R1/R2 | 2.47 | 1.90 – 3.22 | <0.001 | **2.02** | **1.59 – 2.78** | **<0.001** |
| Unknown | 1.63 | 1.22 – 2.16 | <0.001 | 1.18 | 0.87 – 1.59 | 0.292 |
| Differentiation grade |  |  |  |  |  |  |
| Well | 1 |  |  | ^a^ |  |  |
| Moderate | 0.86 | 0.58 – 1.29 | 0.469 | ^a^ |  |  |
| Poor | 1.70 | 1.14 – 2.55 | 0.009 | ^a^ |  |  |
| Unknown | 1.11 | 0.73 – 1.67 | 0.632 | ^a^ |  |  |
|  |  |  |  |  |  |  |

Cox-regression analysis for survival in patients with iGBC. ^a^ Not significant during forward selection.
